# Supplementary material for: The N-Terminal Region of the Polo Kinase Cdc5 Is Required for Downregulation of the Meiotic Recombination Checkpoint
Source: Cells. 2021 Sep 27;10(10):2561. doi: 10.3390/cells10102561 (PMC8533733; doi:10.3390/cells10102561)
Supplement: Supplementary file 1 [file cells-10-02561-s001.zip › cells-1386331-supplementary/cells-1386331-supplementary.pdf]

SUPPLEMENTAL INFORMATION

The N-terminal region of the polo kinase Cdc5 is required for downregulation of the meiotic recombination checkpoint

Sara González-Arranz[1, #], Isabel Acosta[1, #], Jesús A. Carballo[3], Beatriz Santos[1,2] and Pedro A. San-Segundo[1,*]

[1]Instituto de Biología Funcional y Genómica (IBFG). Consejo Superior de Investigaciones Científicas (CSIC) and University of Salamanca. 37007-Salamanca, Spain
[2]Departamento de Microbiología y Genética. University of Salamanca. 37007-Salamanca, Spain.
[3]Department of Cellular and Molecular Biology. Centro de Investigaciones Biológicas Margarita Salas. Consejo Superior de Investigaciones Científicas (CSIC). 28040-Madrid, Spain

[#]These authors contributed equally to this work
*Correspondence: pedross@usal.es

Supplemental Figures (Figures S1-S2)
Table S1 (Plasmids list)
Table S2 (Strains list)
Table S3 (Strains used in Figures)
Table S4 (Antibodies list)

Figure S1

A

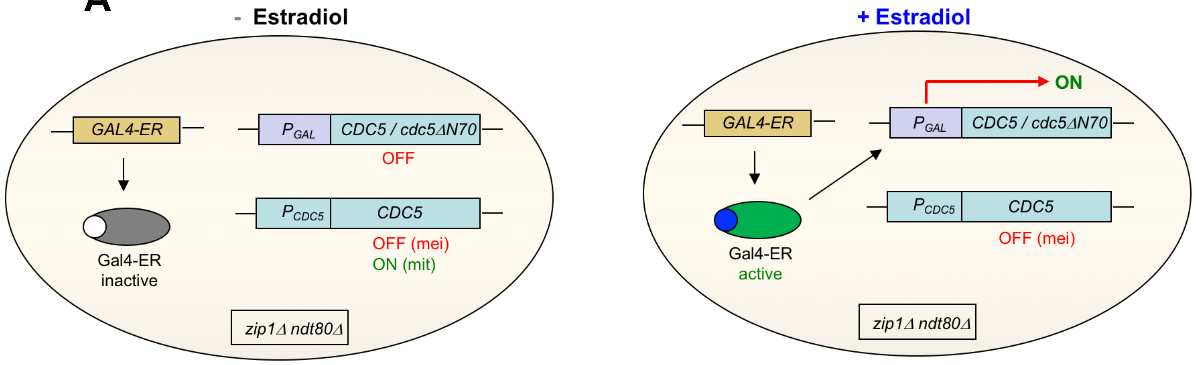

B

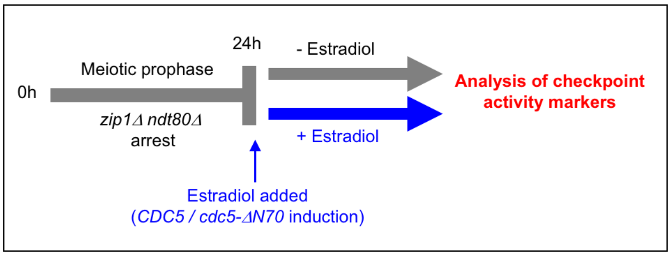

Figure S1. The *CDC5*-inducible system by estradiol (*CDC5-IN*).
(A) Schematic representation of the *CDC5-IN* system used to explore the impact of Cdc5 or Cdc5-ΔN70 on the inactivation of the meiotic checkpoint induced by *ZIP1* deficiency. The strains utilized carry *ZIP1* deletion; therefore, the checkpoint is triggered, and also carry *NDT80* deletion; therefore, they arrest permanently in prophase I. The absence of Ndt80 also prevents the meiotic expression of the endogenous *CDC5* gene. These strains also harbor a version of the Gal4 transcriptional inductor fused to an estradiol receptor (*GAL4-ER*). In the absence of estradiol, Gal4-ER is inactive, but upon estradiol addition it activates the *GAL1* promoter (P_{GAL}). Besides the endogenous *CDC5* gene driven by its own promoter (P_{CDC5}), the strains also contain an additional copy of the *CDC5* gene (or *cdc5-ΔN70*) controlled by the P_{GAL} promoter. Although Cdc5 is essential for viability, the presence of the endogenous P_{CDC5}-*CDC5* gene supports mitotic growth (mit). However, *ndt80Δ* meiotic cells (mei) in the absence of estradiol (left) express neither P_{CDC5}-*CDC5* nor P_{GAL}-*CDC5/cdc5-ΔN70* genes. Addition of estradiol to the medium (right) triggers P_{GAL}-*CDC5/cdc5-ΔN70* expression allowing us to study exclusively the effect of Cdc5 (or Cdc5-ΔN70) without interference from cell cycle progression or from the impact of other targets of Ndt80.
(B) The scheme depicts the experimental setup employed in Figure 5 to analyze checkpoint activity with the *CDC5-IN* system.

Figure S2

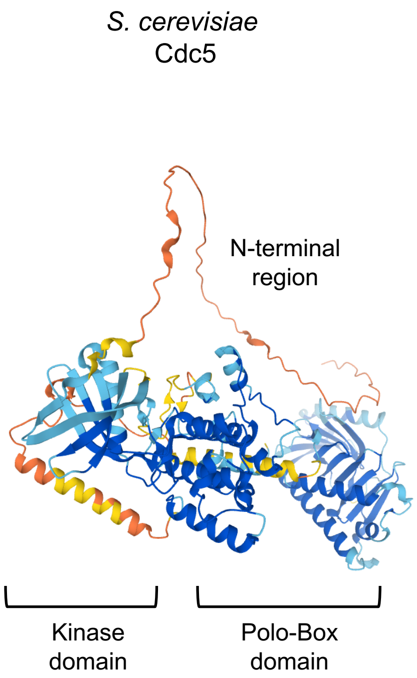

Figure S2. Predicted structure of Cdc5 and PLK1.

Structure prediction of polo-like kinases from budding yeast (Cdc5) and human (PLK1) using the high-accurate AlphaFold DB tool (https://alphafold.ebi.ac.uk/). The largely unstructured N-terminal domain of both proteins appears in orange color.

Table S1. Plasmids.

Plasmid	Vector	Relevant parts	Source/Reference
pRS426	-	*2μ URA3*	(Christianson et al., 1992)
pJC29	pRS426	*2μ URA3 HA-CDC5*	(Jaspersen et al., 1998)
pSS122	pRS426	*2μ URA3 HA-cdc5-ΔN70*	This work
pSS252	pSK+	*HA-CDC5*	This work
pSS253	pSK+	*HA-cdc5-ΔN70*	This work
pSS254	pSK+	*HA-CDC5-natMX4*	This work
pSS255	pSK+	*HA-cdc5-ΔN70-natMX4*	This work
pSS308	pRS426	*2μ URA3 cdc5-T23A*	This work
pSS309	pRS426	*2μ URA3 cdc5-T70A*	This work
pSS310	pRS426	*2μ URA3 cdc5-S18A*	This work
pSS311	pRS426	*2μ URA3 cdc5-T29A*	This work
pSS359	pSK+	*HA-cdc5-db7A-natMX4*	This work
pSS360	pRS426	*2μ URA3 HA-cdc5-db7A*	This work
pMJ998 (pSS356)	pBR322	*natMX4—P_{GPD1}-GAL4(1-848)ER—P_{GAL1}-CDC5*	M. Lichten
pSS425	pBR322	*natMX4—P_{GPD1}-GAL4(1-848)ER—P_{GAL1}-cdc5-ΔN70*	This work

Christianson, T.W., R.S. Sikorski, M. Dante, J.H. Shero, and P. Hieter. 1992. Multifunctional yeast high-copy-number shuttle vectors. *Gene*. 110:119-122.

Jaspersen, S.L., J.F. Charles, R.L. Tinker-Kulberg, and D.O. Morgan. 1998. A late mitotic regulatory network controlling cyclin destruction in *Saccharomyces cerevisiae*. *Mol Biol Cell*. 9:2803-2817.

Table S2. ***Saccharomyces cerevisiae*** **strains**

**Strain**	**Genotype***	**Source**
DP421	*MAT***a***/MAT*α *leu2-3,112 his4-260 thr1-4 trp1-289 ura3-1 ade2-1 lys2ΔNheI*	PSS Lab
DP422	DP421 *zip1Δ::LYS2*	PSS Lab
DP1094	DP421 *HA-CDC5-natMX4*	This work
DP1095	DP421 *HA-cdc5-ΔN70-natMX4*	This work
DP1115	DP421 *HA-CDC5-natMX4 zip1Δ::LYS2*	This work
DP1116	DP421 *HA-cdc5-ΔN70-natMX4 zip1Δ::LYS2*	This work
DP1489	DP421 *HA-cdc5-db7A-natMX4*	This work
DP1490	DP421 *HA-cdc5-db7A-natMX4 zip1Δ::LYS2*	This work
DP1711	*DP421* P_{CDC5}*-CDC5—GAL4(ER)—natMX4—*P_{GAL1}*-CDC5 3MYC-SWE1 zip1Δ::LYS2 ndt80Δ::LEU2*	This work
DP1711	*DP421* P_{CDC5}*-CDC5—GAL4(ER)—natMX4—*P_{GAL1}*-cdc5-ΔN70 3MYC-SWE1 zip1Δ::LYS2 ndt80Δ::LEU2*	This work
JC34	(W303) *MAT***a** *ura3-1 leu2-3,112 his3-11 trp1-1 ade2-1 can1-100 bar1Δ::hisG cdc5-1*	D. Morgan

*All strains, except JC34, are diploids isogenic to BR1919 and homozygous for the indicated markers. DP421 is a *lys2* version of the original BR1919-2N (Rockmill and Roeder, 1990).

Rockmill, B., and G.S. Roeder. 1990. Meiosis in asynaptic yeast. *Genetics*. 126:563-574.

Table S3. Strains and plasmids used in Figure panels

Please, refer to Table S1 and Table S2 for plasmid details and full strain genotypes.

Figure 1B and 1D

Relevant genotype	Strain name / Plasmid name
wild type	DP421 / pRS426
zip1Δ	DP422 / pRS426
zip1Δ OE-CDC5	DP422 / pJC29
zip1Δ OE-cdc5-ΔN70	DP422 / pSS122
zip1Δ OE-cdc5-db7A	DP422 / pSS360

Figure 1C

Relevant genotype	Strain name / Plasmid name
cdc5-1	JC34 / pRS426
cdc5-1 OE-CDC5	JC34 / pJC29
cdc5-1 cdc5-ΔN70	JC34 / pSS122

Figure 2A and 2B

Relevant genotype	Strain name / Plasmid name
zip1Δ	DP422 / pRS426
zip1Δ OE-CDC5	DP422 / pJC29
zip1Δ OE-cdc5-S18A	DP422 / pSS310
zip1Δ OE-cdc5-T23A	DP422 / pSS308
zip1Δ OE-cdc5-T29A	DP422 / pSS311
zip1Δ OE-cdc5-T70A	DP422 / pSS309

Figure 3

Relevant genotype	Strain name
wild type	DP1094
cdc5-ΔN70	DP1095
cdc5-db7A	DP1489

Figure 4

Relevant genotype	Strain name
wild type	DP1094
zip1Δ	DP1115
zip1Δ cdc5-ΔN70	DP1116
zip1Δ cdc5-db7A	DP1490

Figure 5

Relevant genotype	Strain name
zip1Δ ndt80Δ CDC5-IN	DP1711
zip1Δ ndt80Δ cdc5-ΔN70-IN	DP1801

Table S4. Primary antibodies

Antibody	Host and type	Dilution	Source / Reference
Hop1-T318-ph	Rabbit polyclonal	1:1000	(Penedos et al., 2015)
H3-T11-ph	Rabbit polyclonal	1:2000	Abcam<br>ab5168
Pgk1 (22C5D8)	Mouse monoclonal	1:5000	Molecular Probes<br>459250
Red1	Rabbit polyclonal	1:1000	(Smith and Roeder, 1997)
Mek1	Rabbit polyclonal	1:1000	(Ontoso et al., 2013)
Ndt80	Rabbit polyclonal	1:5000	(Benjamin et al., 2003)
Cdc5	Goat polyclonal	1:1000	Santa Cruz Biotechnology<br>sc-6733

Benjamin, K.R., C. Zhang, K.M. Shokat, and I. Herskowitz. 2003. Control of landmark events in meiosis by the CDK Cdc28 and the meiosis-specific kinase Ime2. *Genes Dev*. 17:1524-1539.

Ontoso, D., I. Acosta, F. van Leeuwen, R. Freire, and P.A. San-Segundo. 2013. Dot1-dependent histone H3K79 methylation promotes activation of the Mek1 meiotic checkpoint effector kinase by regulating the Hop1 adaptor. *PLoS Genet*. 9:e1003262.

Penedos, A., A.L. Johnson, E. Strong, A.S. Goldman, J.A. Carballo, and R.S. Cha. 2015. Essential and checkpoint functions of budding yeast ATM and ATR during meiotic prophase are facilitated by differential phosphorylation of a meiotic adaptor protein, Hop1. *PLoS One*. 10:e0134297.

Smith, A.V., and G.S. Roeder. 1997. The yeast Red1 protein localizes to the cores of meiotic chromosomes. *J Cell Biol*. 136:957-967.
